# Simultaneous Dislocation of Both Femurs

**Published:** 1846-04

**Authors:** 


*Simultaneous Dislocation of both Femurs.*—In the *Gazetta Toscana della Scienze Medicofisiche*, a case of simultaneous dislocation of both femurs is detailed. A peasant, thirty years of age, was thrown violently to the ground out of his cart. He could not rise, even with assistance, and experienced severe pain in the right flank. He was immediately conveyed to the hospital, and M. Andreini discovered a dislocation of the right femur upwards and outwards, which was reduced four days afterwards. At the time of admission something abnormal was noticed in the left limb, but as the patient did not complain of pain, and said there was always something wrong with it, and the bone was found not to be broken, no further notice was taken of it at the time. After the right femur had been reduced, it was found impossible to bring the legs together, and the left appeared longer than natural, and turned outwards. It then appeared that, although there still was no pain, the left femur was dislocated downwards and inwards; the deformity spoken of by the patient merely consisted in a slight enlargement of the knee-joint. The dislocation was accordingly reduced, and the patient completely recovered.—*Ibid.*

